# Eosinophilic Angiocentric Fibrosis of the Nasal Cavities: A Report of an Uncommon Lesion with Emphasis on the Etiology and Differential Diagnosis

**DOI:** 10.3390/medicina58070865

**Published:** 2022-06-28

**Authors:** Jessica Farina, Giuseppe Broggi, Carmelo Federico, Magda Zanelli, Andrea Palicelli, Rosario Caltabiano

**Affiliations:** 1 Department of Medical and Surgical Sciences and Advanced Technologies “G.F. Ingrassia”, Anatomic Pathology, University of Catania, 95123 Catania, Italy; jessicafarina2693@gmail.com (J.F.); rosario.caltabiano@unict.it (R.C.); 2 Otorhinolaryngology Unit, Casa di Cura Gibiino, 95128 Catania, Italy; carmelo.federico@libero.it; 3 Pathology Unit, Azienda USL-IRCCS di Reggio Emilia, 42123 Reggio Emilia, Italy; magda.zanelli@ausl.re.it (M.Z.); andrea.palicelli@ausl.re.it (A.P.)

**Keywords:** eosinophilic angiocentric fibrosis, IgG4-related systemic disease, differential diagnosis

## Abstract

*Background and Objectives*: Eosinophilic angiocentric fibrosis (EAF) is an indolent but sometimes locally destructive lesion with a predilection for the sinonasal tract. Although it was first described in 1983, its etiology remains unknown. Some authors initially attributed EAF to trauma, hypersensitivity, and/or surgical manipulation, while it has been recently suggested to include EAF within the spectrum of IgG4-related systemic diseases. *Materials and Methods*: We report an uncommon case of idiopathic EAF in a 76-year-old male who developed two bilateral tumefactive masses in the nasal cavities. *Results*: As the histological examination showed a subepithelial proliferation of fibroblasts along with sclero-hyaline fibrosis around small-sized vessels (an “onion skin-like” pattern) and an eosinophils-rich inflammatory infiltrate, a diagnosis of EAF was rendered. The differential diagnosis included granuloma faciale, Wegener’s granulomatosis, and Churg–Strauss syndrome. *Conclusions*: Pathologists should be aware of the possibility that this lesion can be part of the wide spectrum of IgG4-related systemic diseases by performing IgG4 investigations to assess adherence to IgG4-related systemic disease criteria.

## 1. Introduction

Eosinophilic angiocentric fibrosis (EAF) is a benign, rare lesion of unknown etiology. It was first described in 1983 by Holmes and Panje as an “intranasal granuloma faciale” [1] due to its histologic characteristic of concentric fibrosis around small-sized arterioles, resembling the overall morphology of granuloma faciale; only in 1985 was it given its histologic descriptive name by Roberts and McCann [2].

Females are affected more often than males. EAF clinically presents as a tumefactive lesion, more often occurring in the upper respiratory tract, the sinonasal region, and the orbit, frequently causing progressive and prolonged airway obstruction [3,4,5,6,7,8]. Septal disease with the lateral wall, paranasal sinuses, and subglottic area involvement may also be seen [9,10,11,12]. Until now, to the best of our knowledge, just a single case of cutaneous involvement has been described [13] as an asymptomatic nodule. It is quite likely that other cases of EAF affecting uncommon sites have gone unreported. As it often relapses after surgical excision and therapy [4], a long-term follow-up is needed. In 2011, Deshpande et al. [14] casually discovered that a patient with EAF had a dramatic increase in IgG4 serum levels. This “serendipitous” finding raised the question of whether EAF could be an IgG4-related systemic disease (IgG4-RSD). Since then, an elevated serum IgG4 concentration (>135 mg/dL) has often been reported [3] in patients with EAF, in addition to immunohistochemical reactivity for IgG4. A recent systematic review of the literature demonstrated how the relationship between EAF and IgG4-RSD still has to be proven [15] because not all EAF cases are strictly related to IgG4.

Depending on its stage, EAF can histologically exhibit multiple morphological features. In the early stages, eosinophilic vasculitis of the submucosal small-sized vessels, as well as eosinophilic and lymphoplasmacytic infiltrates, are the predominant features. As the disease progresses, fibrosis arises. The late-stage morphology consists of subepithelial thickening due to dense fibrosis and loss of plasma cells and lymphocytes. The deposition of collagen in a concentric lamellar way around the vessels gives the characteristic “onion skin-like” appearance, while eosinophils are prominent. Although immunohistochemistry is not mandatory to diagnose EAF, it is fundamental to relate it to IgG4-RSD [1].

We report a case of a male patient affected by idiopathic EAF, who lacked both elevated IgG4 serum levels and tissue IgG4^+^ plasma cells in the perivascular infiltrate; the possible etiologies and differential diagnoses are also discussed.

## 2. Materials and Methods

**Figure 1:**
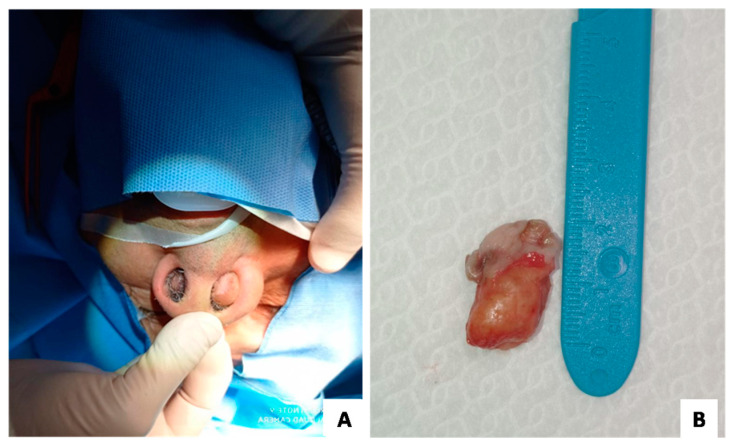
(**A**) Patient shows bilateral tumefactive masses of the nasal cavities. (**B**) On gross examination, a 2-cm polypoid lesion, firm in consistency and grayish in color, that subdues the overlying nasal mucosa, is seen.

A 76-year-old male patient without previous history of nasal obstruction developed two tumefactive masses in both nasal cavities (Figure 1A). No previous history of trauma, hypersensitivity, state of altered immunity, COVID19 infection, and/or surgical manipulation was reported. No significant elevation of autoantibodies was detected on blood tests. The lesions were surgically excised and submitted for histological examination. Gross examination revealed two nodular masses, firm in consistency and grayish in color, each measuring about 2 cm maximum diameter and covered by unaffected nasal mucosa (Figure 1B). Tissue samples were formalin-fixed, paraffin-embedded, and stained with hematoxylin and eosin (H&E). Lesions were immunohistochemically tested with an anti-IgG4 antibody.

## 3. Results

**Figure 2:**
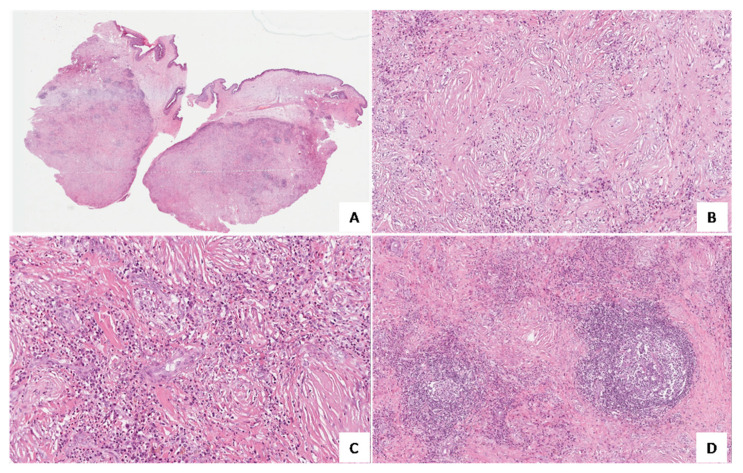
Histological examination. (**A**) Low magnification showing a well-circumscribed, moderately-cellular lesion that submerges the overlying unaffected nasal mucosa (H&E; original magnification 25×); (**B**) Marked stromal sclerohyalinosis with a perivascular “onion skin-like” growth pattern is seen (H&E; original magnification 200×); (**C**) Diffuse eosinophilic inflammatory infiltrate is shown (H&E; original magnification 200×); and (**D**) Plasma cells and lymphocytes are focally arranged in a follicular pattern (H&E; original magnification 150×).

Histological examination of both lesions showed a subepithelial proliferation of bland-looking fibroblasts with an expansive growth pattern and well-circumscribed borders (Figure 2A). As a characteristic finding, sclero-hyaline fibrosis was seen around small-sized vessels, with an “onion skin-like” pattern (Figure 2B). The inflammatory infiltrate was predominantly eosinophilic (Figure 2C), with fewer lymphocytes and plasma cells, focally organized in a follicular pattern (Figure 2D). Based on the morphological features, a diagnosis of “*eosinophilic angiocentric fibrosis*” was rendered. Accordingly, the lesion was immunohistochemically tested with an anti-IgG4 antibody, but no IgG4-positive cells were seen. In addition, IgG4 serum levels were also normal (92 mg/dL). Serologic diagnostic work-up for the patient included anti-proteinase 3, c-ANCA, p-ANCA, and anti-myeloperoxidase, but no positivity was found. The erythrocyte sedimentation rate (ESR) was normal. Full body-computed tomography did not show other localizations of disease. The patient is now healthy without local recurrence of disease after 11 months of follow-up.

## 4. Discussion

EAF is an indolent lesion, sometimes locally destructive and with a predilection for the sinonasal tract. Its etiology is still unclear; it was initially attributed to trauma, hypersensitivity, and/or surgical manipulation, while some authors recently suggested including EAF within the spectrum of IgG4-RSD [1,14,15].

Conventional histopathological features of EAF include perivascular concentric fibrosis (“onion skin-like”) and eosinophils-rich inflammatory infiltrate. However, the histopathology tends to differ depending on the stage of the disease; while in the early stage the eosinophilic vasculitis is patchy, as the disease progresses, fibrosis arises, until it is the only prominent feature in the late stage [1]. Differential diagnosis includes head and neck pseudotumors characterized by fibroblastic proliferations with eosinophil-rich infiltrate, such as granuloma faciale (GF) and granulomatous vasculitis with prominent eosinophilic infiltrate, including Wegener’s granulomatosis, Churg–Strauss syndrome, and Kimura’s disease (Table 1). Roberts and McCann [2] reported a case of EAF associated with GF, suggesting that EAF was a rare mucosal variant of GF, due to its striking overlapping morphology [16,17,18,19,20]. In 2001, the first case of EAF associated with Wegener’s granulomatosis was described [21], emphasizing the fact that increasing associations between EAF, GF, and Wegener’s granulomatosis could explain its etiology, with EAF representing an unusual and exaggerated reaction pattern. The presence of geographic necrosis, necrotizing vasculitis, and granulomatous inflammation support the diagnoses of Wegener’s granulomatosis and Churg–Strauss syndrome, along with positive blood tests for c-ANCA and p-ANCA, respectively, while the lack of the “onion-skin pattern” concentric fibrosis around small-sized vessels supports the diagnosis of GF [22].

In the last few decades, EAF was suggested to be part of IgG4-RSD, due to a casual discovery of high IgG4 serum levels and IgG4^+^ plasma cells in four out of five biopsies [14]. Hallmarks of IgG4-RSD are the presence of tumefactive lesions, dense lymphoplasmacytic infiltrate, fibrosis, and obliterative phlebitis [14]. Histologic criteria to assess whether a lesion can be considered part of the IgG4-RSD spectrum include the presence of the characteristic morphological features, an elevated number of IgG4^+^ plasma cells (more than 50 IgG4^+^ per high power field), and an IgG4^+^:IgG^+^ ratio greater than 40% [1,23]. Crucial histopathological differences between EAF and IgG4-RSD are the lack of obliterative phlebitis and the presence of an eosinophil-rich infiltrate in EAF. Few EAF cases have been reported in the literature with a full evaluation of IgG4-RSD criteria [5,7,11,22,24] and only a few of them have met the criteria to be considered part of IgG4-RSD. Some studies have hypothesized that the similarity between EAF and IgG4-RSD is more striking in the early stages of the disease, while in the later stages it becomes more difficult to show both IgG4^+^ plasma cells in the perivascular infiltrate and high IgG4 serum levels [9,10]. Table 2 summarizes the previously reported cases of EAF and their association with IgG4 serum levels.

In the present case, in which the immunohistochemical tests for IgG4^+^ plasma cells were negative, it was crucial to rely on the conventional EAF histopathological features in order to avoid misdiagnoses and mistreatments. The evaluation of the presence of IgG4^+^ plasma cells is helpful to determine whether the lesion is responsive to glucocorticoid treatment and whether surgery can be avoided, since IgG4-RDs are extremely responsive to medical treatment, but it should not rule out EAF diagnosis when negative.

Recently, an attempt to evaluate the relationship between EAF and IgG4-RSD has been made by reviewing literature and using a validated set of criteria [15]. The result was that a small number of EAF patients met the 2019 ACR/EULAR classification criteria for the diagnosis of IgG4-RSD. Despite all the limitations of this study, the possibility that EAF is not part of the IgG4-RSD spectrum remains and must be kept in mind.

## 5. Conclusions

The present report emphasizes that EAF must be assessed by identifying the classical histopathological findings; however, we would like to emphasize that pathologists should be aware of the possibility that this lesion can be part of the wide spectrum of IgG4-RSD and that they should perform IgG4 investigations to assess the adherence to IgG4-RSD criteria to guarantee the best treatment and outcome for the patient.

## Figures and Tables

**Table 1:** Main differential diagnoses of EAF.

Disease	Usual Sites	Laboratory Tests	Histopathological Features
Eosinophilic angiocentric fibrosis	Upper respiratory tract and orbit	IgG4 serum levels (not specific)	“Onion skin-like” perivascular fibrosis, eosinophil-rich inflammatory infiltrate
Granuloma faciale	Skin (face)	None	Fibrosis and inflammatory infiltrate
Wegener’s granulomatosis	Upper respiratory tract, lungs, and kidneys	c-ANCA	Foreign-body giant cells, geographic necrosis, and granuloma
Churg–Strauss syndrome	Upper and lower respiratory tract, kidney, heart, and gastrointestinal tract	p-ANCA and blood eosinophilia	Fibrinoid necrosis and extravascular granulomas with eosinophil-rich inflammatory infiltrate
Kimura’s disease	Skin (head and neck)	Blood eosinophilia and raised IgE serum levels	Fibrosis and lymphoid aggregates

**Table 2:** Clinical features of the previously reported cases of EAF.

Site	Nasal Region (*n* = 6) [2,9,10,11,14]	Orbital Region, Including Meninges and Ocular Adnexa (*n* = 7) [5,6,8,14,24]	Subglottis (*n* = 1) [12]	Upper Arms and Chest (*n* = 1) [13]
IgG4 serum levels (normal, 8 to 140 mg/dL)	Low (*n* = 1) [6]	Normal (*n* = 3) [5,10,11]	Elevated (*n* = 3) [8,14,24]	Not available (*n* = 8) [2,9,12,13,14]

## Data Availability

No new data were generated in this study.
